# Surveillance of non-muscle invasive bladder cancer using fluorescence in situ hybridization: Protocol for a systematic review and meta-analysis

**DOI:** 10.1097/MD.0000000000014573

**Published:** 2019-02-15

**Authors:** Tianhai Lin, Hongyu Jin, Lina Gong, Ruichao Yu, Sheng Sun, Lu Yang, Peng Zhang, Ping Han, Jingqiu Cheng, Liangren Liu, Qiang Wei

**Affiliations:** a Department of Urology, Institute of Urology; b Key Laboratory of Transplant Engineering and Immunology, West China Hospital; c West China School of Medicine, Sichuan University, Chengdu, Sichuan, China; d Department of Pathophysiology and Molecular Pharmacology, Joslin Diabetes Center; e Massachusetts General Hospital Cancer Center, Harvard Medical School, Boston, MA.

**Keywords:** bladder cancer, FISH, NMIBC, recurrence, surveillance

## Abstract

**Background:**

To evaluate the diagnostic effectiveness and predictive value of fluorescence in situ hybridization (FISH) in the surveillance of non-muscle invasive bladder cancer (NMIBC).

**Methods:**

PubMed/Medline, EMBASE, Web of Science, Ovid, Web of Knowledge, and Cochrane Library will be searched for studies related to the topic. The identification, inclusion, and exclusion flowcharts will be conducted according to preferred reporting items for systematic reviews and meta-analysis guidelines. The identified reports will be critically appraised according to the Newcastle–Ottawa scale, quality assessment of diagnostic accuracy studies-2 and standards for reporting of diagnostic accuracy 2015. Forest plots will be generated to display hazard ratios, sensitivities, and specificities. Pooled estimates with their 95% confidence intervals will be calculated using the bivariate model, the hierarchical summary receiver operating characteristic model and a fixed- or random-effects model.

**Results:**

This study will provide evidence and data to form a comprehensive understanding of the value of FISH in the surveillance of NMIBC.

**Conclusion:**

The diagnostic efficacy of FISH will be affected by post-therapy factors. However, FISH still could facilitate the surveillance of NMIBC owing to its non-invasive feature. This study will improve the clinical decision-making and enlighten the future research of NMIBC.

## 1 Introduction

Bladder cancer is among the most common cancers of the urinary tract.^[1]^ Although the majority of bladder cancers are non-muscle invasive when initially diagnosed, they are at high risk of recurrence and one-third of patients require repeat resections.^[2]^ Patients with non-muscle invasive bladder cancer (NMIBC) are under long-term surveillance since diagnosis. The follow-up regimen includes cystoscopy, imaging, and urine cytology, but 60% of patients relapse within 3 years of initial treatment.^[3]^ Urine fluorescence in situ hybridization (FISH) is a technique that allows the visualization of genetic aberrations of bladder cancer, thus is capable of detecting early stage NMIBC.^[4]^ Additionally, a recent systematic review and meta-analysis conducted by our group prove that FISH has a higher sensitivity compared to cytology for diagnosing upper urinary tract tumors, which are more latent than NMIBC.^[5]^ Over the previous decade, several studies have investigated FISH in the surveillance of NMIBC, to evaluate its efficacy for detecting recurrence and predicting progression.^[6,7]^ However, many of the studies have small sample size and hence less power. The present study is designed to synthesize currently available evidences about the use of FISH in the surveillance of NMIBC, to assess its predictive value for recurrent disease.

## 2 Methods

The protocol has been registered on the international prospective register of systematic reviews (PROSPERO registration number: CRD42019121035. Available at: http://www.crd.york.ac.uk/prospero/). This protocol is conducted according to preferred reporting items for systematic reviews and meta-analyses protocols statement guidelines. The essential protocol amendments will be documented in the full review if applicable. This study was approved by the Ethics Committee of West China Hospital, Sichuan University (Chengdu, China).

### 2.1 Evidence acquisition

Authenticated databases including PubMed/Medline, Embase, Web of Science, Ovid, Web of Knowledge, and Cochrane Library will be extensively searched for articles written in English and published from January 2005 to November 2018.

MeSH words and free words with the following searching strategy: (“FISH” or “fluorescence in situ hybridization”) and (“bladder cancer” or “bladder carcinoma” or “urothelial carcinoma” or “urothelial cancer”) and (“recurrence” or “progression” or “surveillance” or “monitor”) will be used in the literature search. Obvious duplicates will be removed, and studies with inaccessible full texts or categorized as case reports, editorials, reviews, or letters will be excluded as well. Studies qualified for further analysis must meet the following criteria: included more than 15 NMIBC patients; reported primary outcomes including overall survival, progression-free survival, disease free-survival, sensitivity or specificity of FISH, and the control group; and were randomized controlled trials and any observational design, including cross-sectional, case–control, self-control, and cohort designs.

Two independent reviewers will participate in the screening process and analyzed all full texts. If a discrepancy arises, a 3rd reviewer will be summoned to adjudicate the conflict.

### 2.2 Data extraction

The following information was extracted: title, author, nationality, department, ethnicity, study design, age and sex of the patients (both the experimental and control group), enrollment year, and parameters of correlated outcomes. Primary outcomes include overall survival, sensitivity, and specificity, secondary outcomes include progression-free survival and disease free survival. An electric data table containing extracted parameters will be generated from the included articles by 2 individual reviewers simultaneously, and discrepancies will be resolved by a 3rd reviewer.

### 2.3 Quality evaluation

Standard quality evaluation of the included studies will be performed based on following criteria and their respective protocols: the Newcastle–Ottawa scale, quality assessment of diagnostic accuracy studies-2, and standards for reporting of diagnostic accuracy 2015 tools.^[8,9]^

### 2.4 Bias assessment

Funnel plot will be implemented to detect the risk of publication bias when ≥10 studies are qualified for analysis. Otherwise, Begg test and Egger test will be applied using STATA 14.2 (StataCorp, College Station, TX).

### 2.5 Statistical analysis

For diagnostic accuracy tests, data will be extracted to reconstruct a 2 × 2 table, which will be used to calculate sensitivity, specificity, positive predictive value, negative predictive value, odds ratio, and diagnostic likelihood ratios along with the 95% confidence intervals for each study. Then the forest plots will be generated to display sensitivity and specificity estimates using Review Manager 5.3 (The Cochrane Collaboration). To summarize test performance, the bivariate model and the hierarchical summary receiver operating characteristic model will be carried out by metandi (meta-analysis of diagnostic accuracy using hierarchical logistic regression) command in STATA 14.2 (StataCorp).^[10,11]^

For survival outcomes, estimates will be derived and presented in forest plots and reported as hazard ratios with their 95% confidence intervals using Review Manager 5.3 (The Cochrane Collaboration). Heterogeneity will be evaluated using the *I*^2^ test by STATA 14.2 (StataCorp) before the meta-analysis. Then a fixed-effects model will be used if appropriate levels of heterogeneity are present, otherwise, a random-effects model will be applied. If meta-analysis is not appropriate, we will use narrative synthesis and the results will be qualitatively described from clinically comparable studies.

## 3 Discussion

This systematic review will assess the diagnostic accuracy and predictive value of FISH in the surveillance of NMIBC. FISH has been proved to be effective in patients with suspicious bladder cancer and upper urinary tract carcinoma, but whether it remains accurate in the post-therapy scenario is uncertain. A lot of factors are likely to abrogate the efficacy of FISH for detecting NMIBC recurrences, such as wound-healing process, urinary tract infections, and intravesical therapies.^[12]^ However, FISH still could facilitate the surveillance of NMIBC since it is non-invasive and convenient, especially for patients with urinary diversion or urethrostenosis, who are unfit for cystoscopy.^[13]^ Another benefit of applying FISH in the surveillance of NMIBC is it may predict recurrence and progression in the absence of visible tumor, which indicates a more vigilant follow-up schedule is needed.^[14]^ We acknowledge several limitations may present in this study. First, RCTs are estimated to be scarce, which may introduce selection bias. Second, a general standard of interpreting the results of FISH is lacked. Different kits and cut-off thresholds may cause observer bias. Notwithstanding its limitation, this study will provide solid evidence and synthesized data to form a comprehensive understanding of the value of FISH in the surveillance of NMIBC, which will benefit clinicians and researchers who are interested in the management of NMIBC.

## Author contributions

**Conceptualization:** Tianhai Lin, Liangren Liu, Qiang Wei.

**Data curation:** Tianhai Lin, Lina Gong.

**Formal analysis:** Tianhai Lin, Hongyu Jin.

**Funding acquisition:** Jingqiu Cheng, Qiang Wei.

**Investigation:** Hongyu Jin, Lina Gong.

**Methodology:** Lu Yang, Liangren Liu.

**Project administration:** Peng Zhang, Ping Han.

**Software:** Tianhai Lin, Sheng Sun.

**Supervision:** Qiang Wei.

**Writing – original draft:** Tianhai Lin, Hongyu Jin.

**Writing – review and editing:** Tianhai Lin, Ruichao Yu.

Tianhai Lin orcid: 0000-0003-0954-3282.
